# Digital Tracking of Rheumatoid Arthritis Longitudinally (DIGITAL) Using Biosensor and Patient-Reported Outcome Data: Protocol for a Real-World Study

**DOI:** 10.2196/14665

**Published:** 2019-09-26

**Authors:** William Benjamin Nowell, Jeffrey R Curtis, Sandra K Nolot, David Curtis, Shilpa Venkatachalam, Justin K Owensby, Jiat Ling Poon, Amy B Calvin, Carol L Kannowski, Douglas E Faries, Kelly Gavigan, Virginia S Haynes

**Affiliations:** 1 Global Healthy Living Foundation Upper Nyack, NY United States; 2 School of Health Professions University of Alabama at Birmingham Birmingham, AL United States; 3 Eli Lilly and Company Indianapolis, IN United States

**Keywords:** real world evidence, real world data, patients, rheumatoid arthritis, patient-reported outcomes, patient-generated health data, mobile technology, wearable digital technology

## Abstract

**Background:**

Rheumatoid arthritis (RA) is a condition with symptoms that vary over time. The typical 3- to 6-month interval between physician visits may lead to patients failing to recall or underreporting symptoms experienced during the interim. Wearable digital technology enables the regular passive collection of patients’ biometric and activity data. If it is shown to be strongly related to data captured by patient-reported outcome (PRO) measures, information collected passively from wearable digital technology could serve as an objective proxy or be complementary to patients’ subjective experience of RA symptoms.

**Objective:**

The goal of this study is to characterize the extent to which digital measures collected from a consumer-grade smartwatch agree with measures of RA disease activity and other PROs collected via a smartphone app.

**Methods:**

This observational study will last 6 months for each participant. We aim to recruit 250 members of the ArthritisPower registry with an RA diagnosis who will receive a smartwatch to wear for the period of the study. From the ArthritisPower mobile app on their own smartphone device, participants will be prompted to answer daily and weekly electronic PRO (ePRO) measures for the first 3 months.

**Results:**

The study was launched in December 2018 and will require up to 18 months to complete. Study results are expected to be published by the end of 2021.

**Conclusions:**

The completion of this study will provide important data regarding the following: (1) the relationship between passively collected digital measures related to activity, heart rate, and sleep collected from a smartwatch with ePROs related to pain, fatigue, physical function, and RA flare entered via smartphone app; (2) determine predictors of adherence with smartwatch and smartphone app technology; and (3) assess the effect of study-specific reminders on adherence with the smartwatch.

**International Registered Report Identifier (IRRID):**

DERR1-10.2196/14665

## Introduction

As the selection and availability of consumer-grade digital technology to measure biometric and activity outcomes have increased dramatically in recent years, their use in clinical and observational studies have also grown. At a minimum, biosensor technology typically measures heartbeat, activity, and sleep, yet these tools have been used primarily in research in disease states with core symptoms that are clearly directly measurable using such technology (eg, Parkinson’s disease) [1]. An individual’s level of activity and sleep quality can be affected by many other conditions, such as migraine, diabetes, systemic lupus erythematosus, atopic dermatitis, obesity, and arthritis. The ability to observe symptom changes in real time (particularly in response to pharmacotherapy and behavior changes) using mobile biosensor technology has the potential to significantly enhance treatment of chronic disease by enabling more rapid and focused deployment of interventions.

Rheumatoid arthritis (RA) is the most common autoimmune inflammatory arthritis and often results in joint damage that, without adequate treatment over time, may lead to disability, pain, limitations in physical function, and other impairments important to patients [2]. Treating clinicians typically see RA patients at 3- to 6-month intervals. Assessments at clinician visits are necessary, but not enough, to understand the full spectrum of a patient’s clinical state, progression, and the waxing and waning nature of their symptoms. The true severity of pain and flares experienced between visits may not be captured at office visits due to recall bias. To understand the extent of RA disease activity, especially attributes related to pain and stiffness, it is essential to collect patient-reported outcome (PRO) measures in a low-burden, continuous frequency instead of an episodic frequency. PRO measures may include health-related quality of life, physical function, fatigue, sleep, mental health status, work productivity and work activity impairment [3].

PRO measures direct patients to report on their experience and therefore supply unique data for the management of RA. Because PROs are reported directly by patients on paper or electronic questionnaires, they reflect how a patient feels and functions in relation to RA and its therapy [4,5]. Although PRO measures necessitate patient attention and effort, they are also useful for understanding a patient’s subjective experiences. They can help to facilitate clinician-patient communication and shared decision-making to improve the quality of patients’ care, and they can help to identify both common and divergent perceptions of disease activity and treatment effectiveness between clinician and patient. While the advent of smartphone technology has enabled convenient and remote capturing of electronic PRO measures (ePROs) between in-person clinician visits [6,7], a patient must still recall their symptoms over a day- or week-long period. In the quest for additional patient-generated data to complement patients’ reported experiences of physical function, pain, sleep, fatigue, and so on, biometric sensors may play an important role in providing continuously captured objective data (ie, activity, heart rate and sleep hours).

To date, there are few published studies investigating the extent to which biometric data correspond with RA patients’ subjective reports of their symptoms and disease activity. Performance outcome measures (eg, gait, distance traveled, and acceleration) collected passively using smartphone applications have been found to be associated with RA symptoms in studies with small samples ranging from 20-80 participants [8,9]. A recent proof of concept, human factors study explored the experience of 15 subjects who wore an activity tracker daily over 1 week and completed ePRO questions about stiffness, sleep quality, and joint pain in the morning and evening. The investigators reported that initial analyses showed modest correlation between the duration of morning stiffness reported in the ePRO and the level of morning activity tracked by the activity tracker. These investigators are planning a larger trial in a clinical setting with RA subjects receiving medication [10].

In a study of 446 participants, of whom 292 were RA patients, data from daily passive digital measures (ie, Global Positioning System–tracked mobility, mobility radius number, and duration of calls and texts) were collected and associated with PRO measure data (ie, daily pain, patient global health assessment, weekly Health Assessment Questionnaire-II [HAQ-II] [11] and Patient Activity Scale-II [PAS-II] [12]). Text length was most strongly and inversely associated with PROs, including pain, and mobility measures were significantly associated with global assessment, HAQ-II, and PAS-II, but not pain [13]. The Patient Rheumatoid Arthritis Data from the Real World (PARADE) study, launched in 2016, collected ePROs and both active and passive digital data through a smartphone-customized ResearchKit application to measure morning stiffness and fatigue [14,15], but it had disappointing levels of patient engagement. Specifically, of 399 recruited participants, less than half (162; 40.6%) completed one or more study assessments at week 2, and only 45 (11%) remained active in the study by 12 weeks [15,16].

Our exploratory study will expand on these prior studies and examine a larger RA population, incorporating digital measures from a Fitbit Versa smartwatch (chosen based on the type of biometric data captured, water resistance and cost) to assess the value of passively collected digital measures as proxies for RA disease activity and other domains of health that may be affected by RA, as reflected in ePROs. Our study differs from PARADE in the use of smartwatch technology and in the manner of participant recruitment. The PARADE study developed a set of wrist activities to measure range of motion [15], but the activity measurement in our study will be passively collected with the smartwatch. Although less sensitive measures are available via the smartwatch, the ability to collect some measure of activity without requiring participant input is an advantage. In addition, PARADE used a broader approach to identify participants in the United States via targeted digital marketing on social media platforms including Facebook, Twitter, and HealthUnlocked. They also made study information available to Facebook users who followed CreakyJoints. In contrast, our study will enroll patients exclusively from members of ArthritisPower, a CreakyJoints-affiliated research registry in the United States. We believe working within an existing population that is already oriented to sharing ePROs will result in greater participant retention. Unlike PARADE, there will be no randomization into groups based on data sharing with the participant during our study, as all participants will be able to view the same amount of their own data throughout. Additionally, PARADE was conducted entirely via app, with no financial incentives and human interaction. In our study, participants will receive compensation based on specific milestones, and members of the research team will monitor their data in real time and, under certain criteria, contact participants when missing data patterns suggest nonadherence to protocol or difficulty in using the technology. Real-time data monitoring offers the opportunity to clarify protocol procedures directly with individual participants.

Specifically, this study seeks to evaluate the potential relationship between passively collected digital measures related to activity, heart rate, and sleep, collected from a smartwatch, with ePROs related to pain, fatigue, physical function, RA disease activity and flare in participants with RA over the 3-month main study period. In the lead-out period, participants’ use of smartwatch technology will continue to be observed for another 3 months.

The secondary objectives of this study are to: (1) examine the variability of measurement of data derived from digital measures; (2) assess the test-retest reliability and both the convergent and discriminant validity of the digital measures; and (3) determine predictors of adherence with the technology, both with the app and smartwatch.

Exploratory objectives of this study include: (1) evaluating the effect of reminders on adherence with smartwatch use by comparing engagement during the main study period (months 1-3) and lead-out period (months 4-6); and (2) identifying the scientific and operational benefits as well as the challenges confronted in characterizing the burden of illness in RA with digital measures to inform future, digital, real-world evidence studies.

## Methods

### Research Design

#### Overview

This is an ancillary study conducted within the ArthritisPower registry infrastructure. ArthritisPower was jointly developed by the nonprofit Global Healthy Living Foundation (GHLF), its associated CreakyJoints arthritis patient community, and rheumatology researchers at the University of Alabama at Birmingham (UAB) [17,18], and funded through a Patient-Centered Outcomes Research Institute Award (Contract Number PPRN-1306-04811). ArthritisPower currently has over 18,000 consenting participants, about half of whom report a physician diagnosis of RA. As part of their membership in the ArthritisPower registry, participants have downloaded the ArthritisPower app used for ePRO measures collection. This ancillary study, sponsored by Eli Lilly and Company, will collect ePRO measures from RA participants via a customized, study-specific user flow within the ArthritisPower app while measures of activity, heart rate, and sleep will be collected passively using the smartwatch.

The DIGITAL Tracking of rheumatoid Arthritis Longitudinally (DIGITAL) study includes a 10- to 14-day lead-in period, 12-week main study period, and 12-week lead-out period (Figure 1).

#### Lead-In Period

Prior to receiving their smartwatch, invited participants will successfully complete a 14-day lead-in period during which they are required to meet the following inclusion criteria: (1) complete two daily ePRO measures, specifically the single-item Pain and Fatigue numeric rating scales (NRS), on at least 10 of the 14 days; and (2) complete two weekly core sets of ePRO measures (Table 1), over the 14-day period. Those who do not meet these requirements will be offered a single opportunity to repeat the lead-in period and subsequently qualify for the remainder of the DIGITAL study. This initial phase will serve to acclimate participants to regular data collection and provide some assurance that they are willing and able to take part in and complete study requirements over the 3-month main study period.

**Figure:**
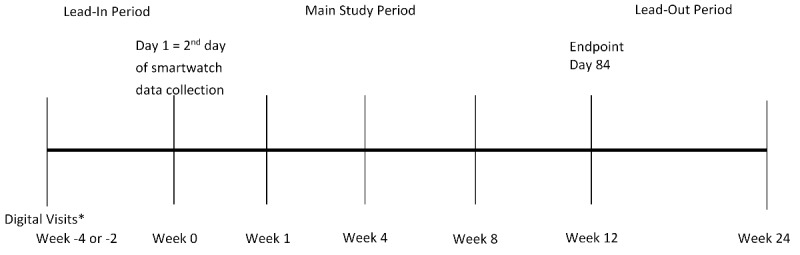
Overall study design. *Active data collection of 2 daily questionnaires begins 2-4 weeks prior to receipt of smartwatch and continues throughout the study. Other electronic patient-reported outcomes measures will be collected weekly. Digital data from the smartwatch will be collected passively.

**Table 1:** Variables and measures.

Categories and variables (frequency/source)			Definition
**Demographic and baseline clinical characteristics (once at registration/ArthritisPower)**			
	Age	Date of birth	
	Gender	Male or female	
	Race	American Indian or Alaskan Native, Asian, Black or African American, Native Hawaiian or other Pacific Islander, Caucasian, multiple race, RTA^a^	
	Hispanic ethnicity	No, unknown, yes	
	Zip code	5-digit US postal code	
	Condition(s)	Rheumatoid arthritis	
	Years since RA^b^ diagnosis	—^c^	
	Rheumatologist name	National Provider Identifier lookup by city, state	
	Height	—	
	Weight	—	
	Current medications, supplements, vitamins, over the counter, and other nonprescription remedies	DMARD^d^ medication class for all medications taken for the treatment of RA. This attribute serves as confirmation of self-reported RA as well as a baseline covariate.	
	Telephone number (cellular)	—	
	Preference for email versus text notifications and reminders	—	
	Typical work schedule	—	
	Typical sleep schedule	—	
**ePRO^e^ measures**			
	Pain, single-item NRS^f^ (daily/ArthritisPower)	0 (no pain) - 10 (pain as bad as it could be) at 0.5 intervals	
	Fatigue, single-item NRS (daily/ArthritisPower)	0 (no fatigue) - 10 (worst possible fatigue) at 0.5 intervals	
	PROMIS-CAT^g^ Fatigue (weekly/ArthritisPower)	0-100 t-score; 0 - <55 (within normal limits), 55 - <60 (mild), 60 - <70 (moderate), ≥70 (severe)	
	PROMIS-CAT Pain Interference (weekly/ArthritisPower)	0-100 t-score; 0 - <55 (within normal limits), 55 - <60 (mild), 60 - <70 (moderate), ≥70 (severe)	
	PROMIS-CAT Physical Function (weekly/ArthritisPower)	0-100 t-score; ≥55 (within normal limits), 40 - <55 (mild), 30 - <40 (moderate), <30 (severe)	
	PROMIS-CAT Satisfaction with Participation in Discretionary Social Activities (weekly/ArthritisPower)	0-100 t-score; ≥55 (within normal limits), 40 - <55 (mild), 30 - <40 (moderate), <30 (severe)	
	PROMIS-CAT Sleep Disturbance (weekly/ArthritisPower)	0-100 t-score; 0 - <55 (within normal limits), 55 - <60 (mild), 60 - <70 (moderate), ≥70 (severe)	
	OMERACT^h^ RA Flare (weekly/ArthritisPower)	0 (low) - 50 (high)	
	Godin Leisure-Time Physical Activity Questionnaire (weekly/ArthritisPower)	0-23 (insufficiently active), ≥24 (active)	
	Adherence to ePRO measure completion (daily or weekly/ArthritisPower)	Ratio of completed ePROs to number of required ePROs prior to discontinuation or end of study period	
	Persistence with ePRO measure completion (daily or weekly/ArthritisPower)	Days until first incomplete or missing ePRO within study period	
**Passively collected biosensor data (continuous [if smartwatch is synced every <5 days]/Fitabase)**			
	Activity	Steps (minute, hour, day) Activity intensity (minute, hour, day) Distance (day) - units = miles Energy expenditure (minute, hour, day) Metabolic Equivalents (minute)	
	Activity-derived variables	Time walking per day (minutes) Time in activity intensity categories per day (minutes) Active time (minutes) Aerobic time (minutes)	
	Heart rate	Beats per minute (minute, day)	
	Heart rate–derived variables	Time in heart rate zone of interest based on exercise charts	
	Sleep	Time sleeping in last 24 hours (minute, day)	
	Sleep-derived variables	Time in light, deep, and REM^i^ sleep and time to sleep onset, time awake and other derived variables (day)	
	Adherence to wearing and syncing smartwatch	Ratio of days with smartwatch data to number of days during study period prior to discontinuation or end of study period	
	Persistence with wearing and syncing smartwatch	Days until first day without any smartwatch data in Fitabase	

^a^RTA: refuse to answer.

^b^RA: rheumatoid arthritis.

^c^Not applicable.

^d^DMARD: Disease-Modifying Antirheumatic Drug.

^e^ePRO: electronic patient-reported outcome.

^f^NRS: numeric rating scale.

^g^PROMIS-CAT: Patient-reported outcome measurement information system-computer adaptive testing.

^h^OMERACT: Outcome Measures in Rheumatology.

^i^REM: rapid eye movement.

#### Main Study Period

Upon successful completion of the lead-in period, participants will receive a Participant Kit that will contain a smartwatch, on-boarding and training materials, and instructions to access study resources such as frequently asked questions, training videos, and research team contact information. Participants will receive reminders as smartphone lock-screen notifications and as emails to sync their smartwatch data and complete their ePROs during the subsequent 84 days. To avoid sending extraneous notifications to those participants who are consistent in smartwatch data download, syncing, and ePRO completion, ArthritisPower will use the daily Fitbit and ePRO data to provide targeted text, email, and phone follow-up reminders, as well as support to participants whose data are missing for one or more days.

#### Lead-Out Period

Following the main study period is a three-month lead-out period, during which no reminders to sync the smartwatch will be sent and no ePRO collection will be prompted beyond normal monthly reminder emails that are sent to all participants in the ArthritisPower registry. The purpose of the lead-out period is to assess the effect of reminders on the main study, observe the attrition in smartwatch use, and assess any changes in smartwatch measures when not actively solicited.

#### Pilot

The study will begin by enrolling a pilot cohort of 10-20 participants who will provide feedback about their experience during the lead-in period and initial setup for the main study period. Participant feedback during the pilot will allow for operational adjustments to be made prior to the larger study start. Participants will provide feedback via emailed questionnaires and phone calls on various logistical aspects, including receiving and completing ePRO measures via the smartphone app, getting a shipped package (Participant Kit) containing the smartwatch, and setting up the smartwatch. This feedback will be used to adjust the smartwatch provisioning process as needed. Any major proposed adjustments would be submitted to the Institutional Review Board for review and approval prior to implementation.

### Study Population

GHLF will send eligible members of the ArthritisPower research registry an invitation to participate in this study. Participants within the ArthritisPower registry are eligible to join this study if they provide informed consent and meet each of the inclusion criteria listed in Textbox 1.

### Compensation

Participants who complete all activities for the first 4 weeks of the main study period will receive a US $25 gift card, and those who complete all activities for all 12 weeks of the main study period will receive an additional US $50 gift card.

### Data Collection

Participants who successfully complete the 14-day lead-in period will be issued a smartwatch that will be used to collect digital measures of activity, heart rate, and sleep. The participants are not required to return their smartwatch at the end of the study. Day 0 of the study is the date that the first smartwatch data will be observed from the participant. The following day is Day 1 for analysis purposes, as it will constitute a full 24-hour period of data collection.

### Variables and Measures

Table 1 presents the study variables and their operational definitions. The t-score used to measure the Patient-reported outcome measurement information system-computer adaptive testing (PROMIS-CAT) is a standardized score based on the overall (healthy) US population where 50 is the average (mean), which allows us to see how much above or below (ie, number of standard deviations) a person’s PROMIS-CAT ePRO deviates from the mean.

### Data Workflow

GHLF will send eligible members of the ArthritisPower research registry an invitation to participate in this study. Once enrolled, participants will provide daily and weekly survey data to be stored in the ArthritisPower database. During the main study period, participants will be equipped with preconfigured Fitbit accounts linked by GHLF to the Fitabase platform. The Fitabase platform will stream the participants’ activity metrics directly from the Fitbit cloud. This will allow the research team at GHLF and UAB to monitor participant sync and charge activity and send participation reminders. Accessing Fitabase via an exposed application programming interface enables automation of this process.

Identifying information will be collected separately from survey responses and digital measures of activity, heart rate, and sleep to protect participant privacy and maintain a deidentified data set. All identifying information collected will be handled by internal ArthritisPower research staff, as per guidelines specified in ArthritisPower protocol. All data collected through this study will be analyzed, stored, and collected by ArthritisPower staff at GHLF and UAB. Individual identities will only be used by ArthritisPower staff at GHLF and UAB to send smartwatches and follow up with participants to troubleshoot problems with data collection or syncing. Any results will be reported in a deidentified or aggregated form. All information will be stored and protected in password protected files and can only be accessed by the ArthritisPower research team. The only participant-level data that will be shared with Eli Lilly and Company is the deidentified smartwatch data to enable greater understanding of passively collected smartwatch data. It will not be possible for the research team at Eli Lilly and Company to contact participants for this study. Any identifying information collected will not be included in any notes or report documents used by the team. Data flow throughout the study is outlined in Figure 2.

**Inclusion criteria for the DIGITAL study.**

- Age 19 or older
- US resident
- Self-reported diagnosis of rheumatoid arthritis
- Registered a valid email address with ArthritisPower
- Currently being seen by a US rheumatologist
- Currently taking at least one conventional synthetic or targeted disease-modifying antirheumatic drug for rheumatoid arthritis, but not baricitinib
- Own a smartphone (iPhone 4S and later or Android 4.3 and later) to which they have downloaded the ArthritisPower app
- Are willing to contribute daily and weekly ePROs for up to 98 days, and health activity tracker data for at least 84 days
- Are willing to wear the smartwatch while sleeping
- Will not be out of internet access (Wi-Fi or mobile data) for 4 or more consecutive days during the study
- Successfully complete the lead-in period
- Are willing to be contacted by e-mail or phone by a study coordinator if they fail to adhere to the study protocol

**Figure:**
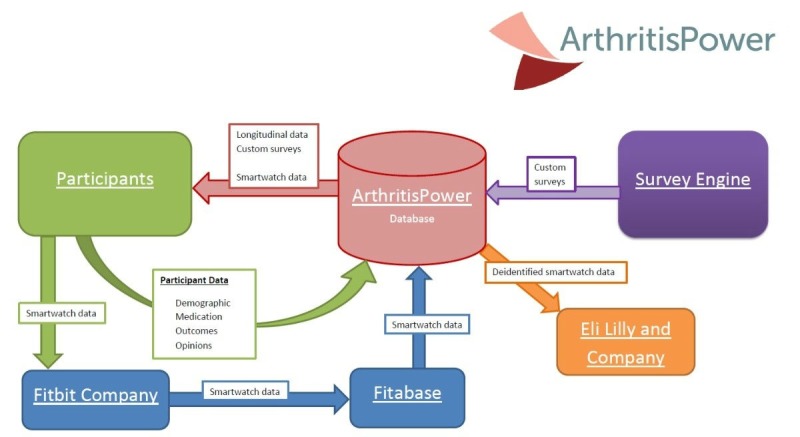
Study data workflow.

### Analyses

#### Primary Analyses: Agreement Between ePRO and Passively Collected Digital Measures

The primary objective of this study is to quantify the agreement between passively collected digital measures (eg, activity) and ePRO data (eg, pain NRS scores). Descriptive statistics will summarize the demographics and baseline characteristics for all enrolled participants. The primary analysis will be a descriptive summary of the correlation between passively collected biosensor data and ePRO data. See Table 1 for a list of the ePRO and passively collected digital measures. For ePROs assessed weekly, multiple summary measures of the passive measures will be created over the corresponding weekly timeframe. Summaries to be explored include the average over the time period, trends, most recent, minimum, maximum, variation, and transformations in the data. Correlations between the ePRO and passive measures will be quantified using both a simple correlation matrix for each week as well as using repeated measures models over the entire study. Repeated measures models will be implemented using each ePRO as the outcome measure, with time (eg, week as a classification factor), baseline measures, and the various passive measures as potential factors in the model. The starting model will be a simple main effects model, and then a penalized regression model will also be used to optimize model selection, including the potential for 2-way interactions (including with time). This will allow for assessment of changes in correlations over time adjusted for participant level covariates.

For digital measures obtained daily, a repeated measures model will assess the association between the ePRO (outcome) and passive measures over time (with day as the time period of assessment rather than week). Daily ePRO data may lag by a day if it is observed that most participants are responding in the morning. Due to the large number of days in the study, time will be considered a numeric variable in this model.

#### Secondary Analyses

Test-retest reliability will be assessed by examining the correlations between each derived digital measure during time periods when observed values of the related ePROs are stable. This includes, for example, assessing the intraparticipant correlations between weekly averaged daily step counts (or associated derived variables) on weeks when participants report the same level of activity as assessed using the Godin Leisure Time Physical Activity Questionnaire [19].

Random forests, gradient boosting, and penalized regression models incorporating cross-validation will be built for detection and prediction of ePRO defined events (eg, flare, adherence, score changes). Baseline and time-varying factors (ie, ePRO, digital measures, and changes in previous time periods) will be included as potential factors in the analyses. Operating characteristics of models will be compared.

Using the Outcome Measures in Rheumatology (OMERACT) flare PRO [20], a question was added after Q7 (“Are you having a flare now?), “If yes, how long ago did it start?” Possible responses included, “I’m not having a flare at this time, Today, Yesterday, 2 days ago, 3 days ago, 4 days ago, 5 days ago, 6 days ago, 7 or more days ago,” so that this study could define the onset date of any RA flare. Descriptive statistics will summarize the trends in the daily ePRO and passive data over the last 3 days prior to the onset of the flare. To assess whether digital measures can accurately classify participants as having an RA flare or not, Classification and Regression Trees and penalized regression incorporating cross-validation will be used.

#### Interim and Subgroup Analyses

The interim analysis will be conducted after the initial pilot cohort have completed 4 weeks of the study.

### Sample Size and Statistical Methods

We aim to recruit 250 participants who have successfully completed the lead-in period and received the smartwatch to wear for the duration of their participation in the main study and lead-out periods. Assuming at least 75% of the participants complete the majority of the measures, a sample size of 250 will provide at least 80% power to detect correlations between passively collected data via the smartwatch and the actively collected data from ePRO instruments of at least 0.2 and over 90% power to detect correlations of at least 0.3 at any given time point. The analyses will not include any adjustment for multiplicity.

## Results

**Figure 4:**
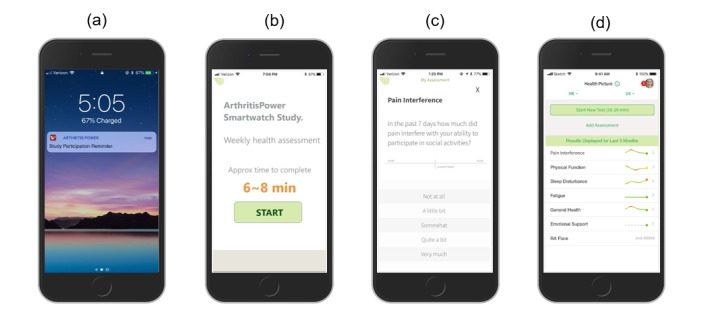
Main study period screen shots: a) Participant receives email and smartphone lock screen notification with reminder to complete daily and weekly assessments; b) Participant is informed of approximate time required to complete daily or weekly assessments so they can start when ready; c) Participant progresses through daily and weekly assessments, including Pain Interference; d) Upon completion of weekly assessment queue, participant sees a Health Picture summary of personal assessment scores.

A user flow was designed, developed, and tested for the lead-in and main study periods of the project. Screen shots displaying the user flow for the lead-in period are shown in Figure 3 and for the main study period in Figure 4. The study was launched in December 2018 with pilot participants. As of February 2019, 17 pilot participants have enrolled in the study, have completed the lead-in period, and have received a smartwatch and begun syncing their data as part of the main study period.

Invitations were sent to 70 eligible ArthritisPower participants, with a total of 17 participants enrolling in the pilot of the DIGITAL Study. Once they completed the lead-in period and successfully started the main study period, pilot participants provided feedback via email, online questionnaire, and phone calls. The research team also examined the ePRO and smartwatch data that had been collected from pilot participants to flag preliminary issues with participant adherence to protocol procedures and missing data. Three issues emerged that were addressed via modification to the operational plan and the app software (underlying user flow for this study).

First, participants appreciated getting daily reminders during the lead-in period and asked that these daily reminders continue throughout the main study period. Second, participants were confused about the difference between their ePRO measures for the week as part of the DIGITAL Study and the regular weekly ePRO measures completed within the standard ArthritisPower registry. This made it difficult for participants to distinguish whether they had completed their DIGITAL Study tasks for the day or week. As a result, we changed the ArthritisPower app user experience for those in the DIGITAL Study so that while ArthritisPower participants are taking part in the DIGITAL Study, their regular weekly ePROs in ArthritisPower will be disabled to avoid confusion and duplication of effort. Third, smartwatch data syncing presented some challenges for participants, with at least one participant in the pilot cohort believing they were syncing data correctly even though data were not appearing to the research team as expected. After troubleshooting, we discovered that the participant had logged in to a prior, personal Fitbit account, which meant data were not captured for analysis in the study during that period of their smartwatch use. Additionally, a few pilot participants said they found the smartwatch band to be uncomfortable and would take it off, sometimes forgetting to put it back on.

**Figure:**
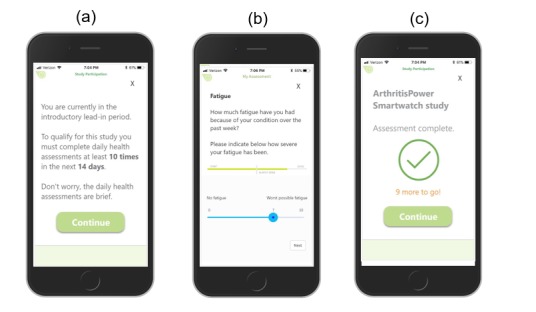
Lead-in period screen shots: a) Participant is presented with introductory screen, reminded of lead-in requirements to be eligible for the main study, and prompted to continue to assessments; b) Participant completes assessments, including daily single-item Fatigue measure; c) Upon completion of assessment queue, participant is reminded of remaining number of sets of assessments to be eligible for the main study.

We had planned several steps to minimize the technical trouble participants might encounter in syncing their smartwatch data and to enable the research team to quickly identify when smartwatch data was not being provided from a participant. First, in the Participant Kit that included the smartwatch, we provided simple set-up instructions on a “Start Here” 8.5”x11” card that included the Fitbit app download, and we included login information with a login email address and password that was unique to each participant. Since we knew in advance the participant’s login information, we could immediately see who was successfully syncing as soon as they set up their smartwatch. Second, in the ArthritisPower app unique user flow for the DIGITAL study, we prompted participants to let us know when they received their Participant Kit so they could be automatically directed to download the Fitbit app. This is scheduled to occur in the app after the lead-in period to inform the research team of the exact date a participant had taken the necessary steps to set up and sync their smartwatch. Finally, to minimize the risk of perpetual missing data from syncing or incomplete ePROs, we created a partially automated case management structure to rapidly identify participants whose data indicated the need for more intensive follow-up intervention, including calls from staff. To address watch band discomfort, the research team will now make alternative bands available to participants on a case-by-case basis when flagged during case management interactions. Importantly, we noted in the pilot cohort smartwatch data that if a participant removed their smartwatch, they did so either for a short period of time (eg, to shower) and put it back on fairly quickly, or for a long time (eg, to charge). As a result, for this study we agreed that at least one minute of activity or data in a given hour would indicate that the participant’s data could be used for analysis.

The DIGITAL study informed consent and study communications with participants were modified to reflect any changes to the initial operational plan following pilot cohort feedback. This exercise gave us greater confidence in participant data collected beyond the pilot cohort. It is expected that all study participants will have completed the main study period by early 2020 with results published in 2021.

## Discussion

The primary objective of this study is to evaluate the relationship between passively collected digital measures related to activity, heart rate, and sleep, collected from a smartwatch, with ePROs related to pain, fatigue, physical function, RA disease activity, and flare among people living with RA over 3 months. Since passively collected data, such as step counts and heart rate, are measured in a more continuous and objective manner than ePROs, there are implications for both research and patient care depending on the strength of the correlation between these two types of data.

For research, remote data collection of trial participants could minimize participant burden and save time and money required for clinical trials due to less frequent clinical visits and fewer staff hours. Among patients, both activity trackers and remote collection of ePROs mitigate recall bias and offer the opportunity for more comprehensive data collection. Our findings may help inform future studies by identifying when ePRO data are necessary to supplement passive data and when they are not. Collecting data during the interim period between clinician visits is important for patients with RA because each day can be different, so tracking variability in how the disease behaves day-to-day may help better assess disease activity. If ultimately accepted by regulators, this could lead to greater precision and thus smaller clinical trials, resulting in more rapid approval of medications for the marketplace. Moreover, this information facilitates a quicker and more continuous stream of real-world data being generated for comparative effectiveness research. As such, the type of approach represented in this protocol might be integrated into a complete, patient-centric digital health solution and bundled with a medication prescription, a so-called Beyond the Pill approach [21]. As a result, patients and clinicians may benefit from new information about therapies that are equally or more effective using data collected outside of a typical clinical setting. It could, for example, inform management of RA patients on combination therapy who are doing well (eg, in remission) and might consider discontinuing use of a drug without negatively affecting disease activity and symptoms.

Other possible benefits for patient care include the potential to identify treatment effectiveness between office visits, detect arthritis flare, accelerate clinical reevaluation, and speed up treatment modification when disease activity appears to be active, but this fluctuates frequently with intermittent flares. Monitoring patients remotely between visits in a manner that minimizes patient burden and does not require that a patient do more than wear a device could dramatically increase the amount and availability of data to inform specialists and primary care physicians about patients in their care, as well as contribute important information to assist with quality reporting and improvement [22].

There are several challenges related to passive data collection and the comparative analyses conducted with such data that we sought to address with this protocol. Wearables are appealing in terms of data collection over more hours each day, yet device selection is critical for patient adherence. Participants are more likely to be adherent for a longer duration when wearing commercial-grade devices that may lack the precision of fit for purpose research-grade devices. Passive data are available only when study participants remember to wear their device and correctly sync or download their data on a regular basis. To minimize the risk of missing data, we created a partially automated case management structure to rapidly identify participants requiring more intensive follow-up intervention. Passive data collection in the context of virtual studies such as this one requires more up-front investment of time to develop patient-centric onboarding materials and digital interfaces, operational plans for regular data review to identify issues with collection, and staffing to address questions that arise during technology onboarding and use.

Second, acceptable wear patterns must be established in advance to determine which downloaded device data actually indicate a participant’s use of the device for analytical purposes. The unit of measurement and associated time boundaries for analysis must be defined and described in the statistical analysis plans or programming requirements. For this study, we agreed that at least one minute of activity or data in a given hour would indicate that the participant’s data could be used for analysis. Comparison of continuous, minute-level data with data received only once daily or weekly makes the time cut-offs even more important and influential on results when lining up the data. Although tempting to seek strong correlations and good fitting models, there is substantial value in uncovering differences between passive real-time data and periodic ePRO measures. The increased variability and periodic divergence may lead to lower correlations, but they are also the reason multimodal assessment and an associated understanding of the different data streams is critical.

Finally, from a generalizability perspective, passive data collection requires participants that have a level of comfort with technology. The PARADE study observed that participants tended to have more years of formal education and be younger than the nationally representative cohort used for comparison [15]. Thus, research populations employing such technology represent a subsample that may not adequately represent important segments of patients with the disease in question.

The findings of this study may ultimately provide guidance for patient-focused drug development among regulators, specifically with the US Food and Drug Administration. A clearer characterization of how different types of patient-generated data describe the patient experience in a complementary fashion may help determine how and where to incorporate each data type into the regulatory decision-making process. Both ePRO measures and biometrics shed light on the patient experience, but on different aspects. Biometrics can continuously capture data like activity, heart rate, and sleep quality in a way that ePROs cannot, but there are aspects of activity, sleep, and other physical, mental, emotional, and social health domains related to biometric readings that biometrics alone are insufficient to understand. Daily and weekly ePRO measures like pain and fatigue that are being examined in this study are ideal for exploring the complementary nature of patient-reported and biometric data.

## Abbreviations

ePRO: electronic patient-reported outcome
GHLF: Global Healthy Living Foundation
HAQ-II: Health Assessment Questionnaire-II
NRS: numeric rating scales
OMERACT: Outcome Measures in Rheumatology
PARADE: Patient Rheumatoid Arthritis Data From the Real World Study
PAS-II: Patient Activity Scale-II
PRO: patient-reported outcome
PROMIS-CAT: patient-reported outcome measurement information system-computer adaptive testing
RA: rheumatoid arthritis
UAB: University of Alabama at Birmingham
